# Novel Technique for Reduction of Ankle Valgus Malunion and Chronic Talus Subluxation during Hindfoot Nailing

**DOI:** 10.1155/2020/8826714

**Published:** 2020-11-16

**Authors:** Matthew Ciminero, Kevin Kang, Amr Abdelgawad

**Affiliations:** Maimonides Medical Center, Brooklyn, NY, USA

## Abstract

In recent years, there has been a significant increase in the incidence and severity of ankle fractures in the elderly. We present a case of an elderly patient with significant medical comorbidities and social issues who sustained an unstable ankle fracture that led to a severe malunion secondary to refusal for surgery and noncompliance with weight-bearing precautions. By choosing an intramedullary fixation method that can be inserted through a biologically advantageous surgical exposure, we can permit early mobilization, create a stable plantigrade foot for ambulation, and alleviate the expected noncompliance with weight-bearing precautions in this patient. Despite debridement of callus, fibular osteotomy, and plate-assisted reduction, a displaced valgus angulation of the ankle joint remained. The nail was removed then reinserted backwards with the bend directed medially instead of its normal lateral position in order to use the bend in the nail as a reduction aid to finalize the reduction. We feel this technique may be of assistance to future surgeons encountering a similar situation.

## 1. Introduction

In recent years, there has been a significant increase in the incidence and severity of ankle fractures in the elderly [1]. As we know well from the hip fracture literature, early mobilization reduces mortality rates [2]. Similarly, decreased mobility worsens preexisting morbidity and decreases the chances of independent living after displaced ankle fractures in the elderly [1]. Treatment of these fractures poses significant challenges to the treating orthopaedic surgeon, as both operative and nonoperative routes lead to complications. Due to medical cocomorbidities and osteoporosis, operative management of unstable ankle fractures may lead to wound healing complications and loss of reduction, while still requiring prolonged periods of nonweight bearing. However, there are numerous publications demonstrating unsatisfactory results of displaced unstable ankle fractures treated with manipulation and cast immobilization in patients over the age of 60, with malunion and nonunion rates from 8 to 19%, wound complications around 10%, and persistent pain in up to 56% of patients [3–7]. Due to the aforementioned poor results, the general consensus in the orthopaedic community is that these fractures are best managed with surgery despite the potential risks of surgery in the geriatric population. In addition to the development of posttraumatic arthritis with malreductions in ankle fractures, there is literature to support that incorrect reduction of ankle fractures leads to biomechanical alterations in sagittal balance for foot loading [8].

We present a case of an elderly patient with significant medical comorbidities and social issues who sustained an unstable ankle fracture that led to a severe malunion secondary to refusal for surgery and noncompliance with weight-bearing precautions. Considering her medical history, social history, and strong desires to have a stable ankle that could immediately weight bear, the decision was made to proceed with a tibiotalocalcaneal (TTC) fusion with a nail. By choosing an intramedullary fixation method that can be inserted through a biologically advantageous surgical exposure, is resistant to axial loading and rotation, and acts as a load-sharing construct, we can permit early mobilization, create a stable plantigrade foot for ambulation [9], and alleviate the known noncompliance with weight-bearing precautions in this patient. In our case, the curve of the nail was used in an off-label fashion as a reduction aid by inserting it backwards to treat a valgus malunion ankle fracture.

## 2. Case Report

**Figure 1:**
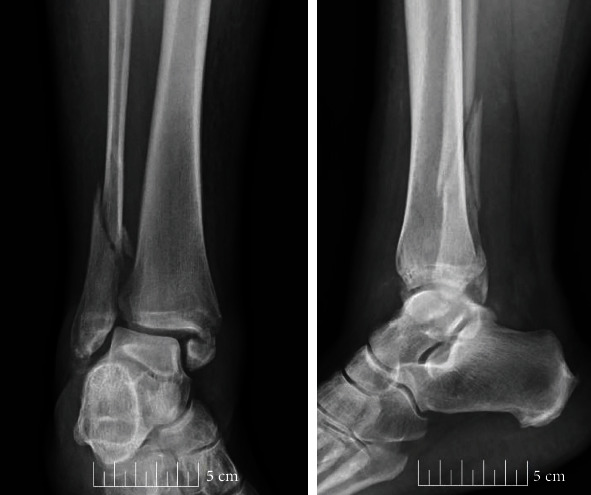
Initial radiographs demonstrating trimalleolar ankle fracture.

**Figure 2:**
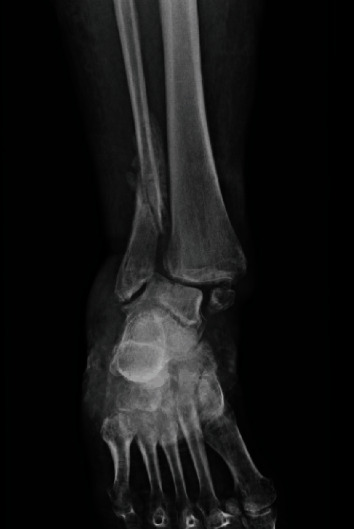
Progression toward chronic ankle deformity and malunion.

A 62-year-old female with a past medical history significant for obesity, chronic obstructive pulmonary disease secondary to tobacco smoking, and a social history significant for alcoholism, prior illicit IV drug abuse, and homelessness presents to the emergency room in September of 2019 after sustaining a supination injury to her right ankle. Radiographs were taken which demonstrate an SER4 trimalleolar fracture (Figure 1) which was closed reduced and temporized in a bivalved short leg cast. After a lengthy discussion with the patient regarding the risks and benefits of surgical and nonoperative management, the patient was adamant that she did not wish to undergo any surgical stabilization of the fracture. She was provided follow-up in the outpatient setting with a walker and recommendations to remain non-weight bearing and was discharged to her shelter. Over the course of the next several months, the patient was noncompliant with weight-bearing precautions in her short leg cast and developed a valgus ankle malunion with severe syndesmotic widening and subluxation of the talus with medial skin tenting (Figure 2). She presented to our hospital in January of 2020 for definitive management secondary to chronic pain, unstable ankle, and difficulty ambulating. Surgical options were discussed with the patient in the form of ankle open reduction and internal fixation, skinny-wire external fixator, ankle fusion with plates and/or screws, and TTC nail fusion. The patient solely wished to undergo a procedure that could allow for early weight-bearing. The decision was made to use a TTC nail.

## 3. Description of the Procedure

After induction of anesthesia, the patient was positioned supine on a radiolucent operating table with the ipsilateral hip bumped and leg elevated on bone foam. Initial radiographs were taken, and motion at the fracture site was assessed with manual reduction techniques. There was minimal to no motion, and the talus remained subluxated with a severe widening of the syndesmosis.

**Figure 3:**
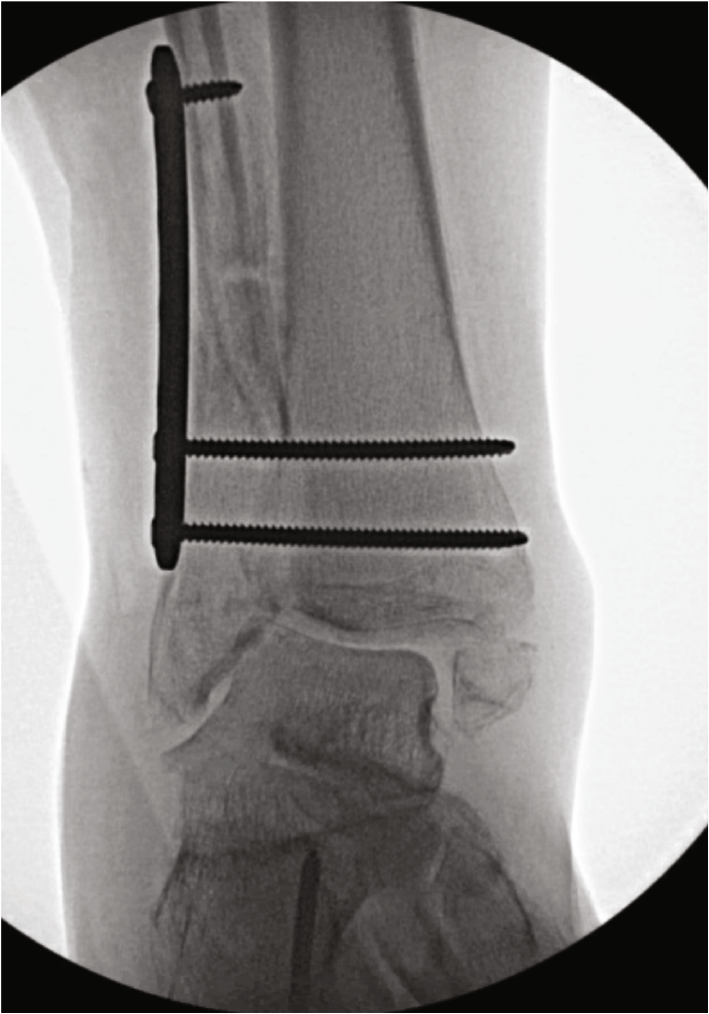
Partial correction of valgus deformity with plate-assisted talar reduction.

Attention was then directed to the fibula. A straight incision was made over the malunited fracture. The previous fracture site was identified, the callus was debrided, and the fibula osteotomized. At this point, there was still minimal talar motion with manual stress. A stout 6-hole LC-DCP plate was applied and fixed proximally and distally. The distal screws were lagged by a technique in a quadricortical fashion to the tibia to assist in straightening the fibula, reduce the syndesmotic widening, and push the talus medially. With this technique, approximately 75% of the talar reduction was achieved in order to get the talus reasonably under the tibia for the passage of the TTC nail. The screws were intentionally placed posteriorly in the tibia to allow for nail passage (Figure 3).

**Figure 4:**
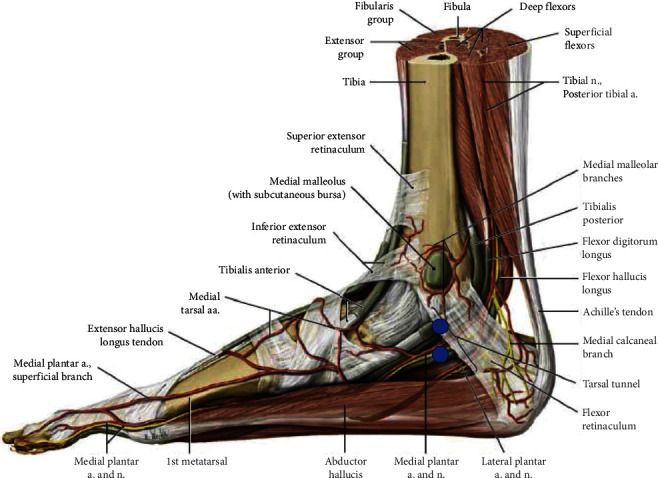
Anatomy of the medial ankle [18].

The TTC nail (T2 Ankle Arthrodesis Nail, Stryker, Kalamazoo, Michigan) was then planned for passage. The foot was held in a plantigrade position, with 5-10 degrees of external rotation. A 2-cm longitudinal incision was made over the plantar aspect of the heel and spread bluntly with a hemostat. The starting point was made in the center of the calcaneus on the calcaneal axial view and centered under the tibial plafond on the sagittal view. The guidewire was passed from the calcaneus, through the talus, and into the tibia using the image intensifier. It was directed medially in order to use the nail to further correct the valgus positioning of the talus. Due to the incomplete talar shift, the guidewire and subsequent reaming in the tibia was at the lateral aspect of the tibial plafond. The pathway was reamed to 13.5mm until sufficient cortical chatter was achieved in order to pass a 12mm × 20cm nail. Many modern generation TTC nails have a built-in valgus bend, obviating the need to medialize the talus, allowing for centralization of the nail within the medullary canal of the tibia, permitting a more lateral start point in the calcaneus to avoid damage to the lateral plantar nerve and vessels, and adjusting for the lateral position of the calcaneus relative to the tibiotalar axis [10–13]. Because of this knowledge of the distal 47 mm of valgus bend built into the nail, the decision was made to invert the nail and insert it backwards so that a varus bend might push the talus further under the tibia. This was successful and fully corrected the remaining deformity. A medial-to-lateral talar screw was placed first using the jig, followed by two proximal tibial locking screws also from medial to lateral. Anatomical studies have demonstrated significantly decreased risk to neurovascular structures with medial to lateral locking screws in the proximal aspect of the nail. Lateral locking options place the anterior tibial artery, peroneal nerves, and extensor digitorum longus and extensor hallucis longus at risk [10]. The standard distal interlocking screws in this third-generation nail are from lateral to medial; however, to use the jig in its new orientation, the screws were placed from medial to lateral. Due to the potential risk of damage to the medial plantar artery and nerve, as well as the tendons passing posteromedial to the medial malleolus, a superficial skin incision was made with blunt dissection down to the bone, and insertion of the trocar to protect these structures (Figure 4).

**Figure 5:**
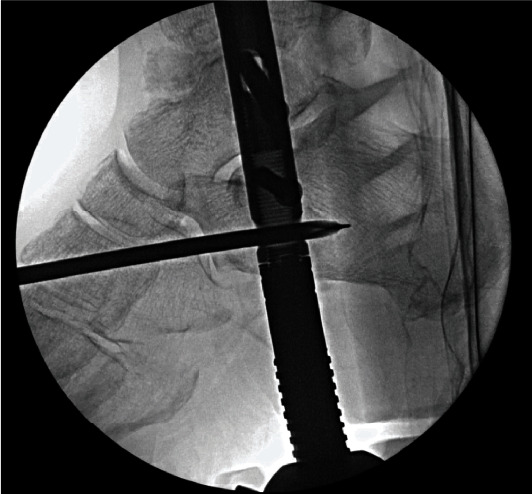
Technique for placement of calcaneal interlocking screw in the tibiotalocalcaneal nail.

**Figure 6:**
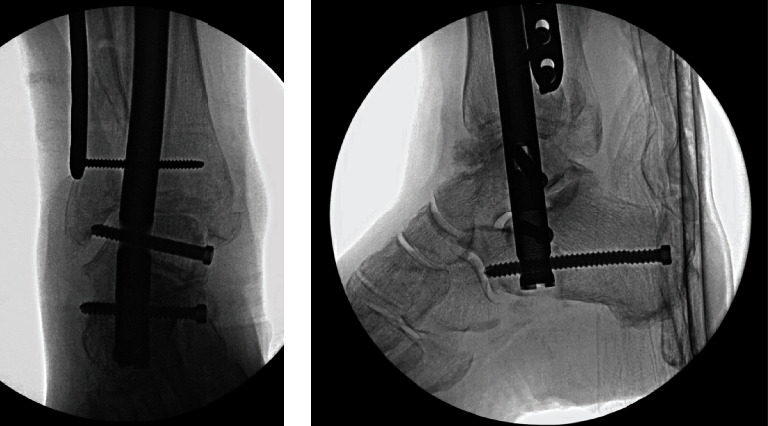
Final implant construct.

The focus was then shifted to a formal fusion of the tibiotalar joint. A small vertical incision was made over the anteromedial ankle. The talus was identified, and the cartilage was debrided using a curette, followed by debridement of the tibial plafond cartilage. Both surfaces were fenestrated with a 2.5 mm drill bit to enhance bleeding surfaces. A rongeur was used to debride the lateral aspect of the medial malleolus, leaving behind a component of the medial malleolus as a medial buttress and increase fusion surface. Synthetic bone graft along with local autograft from the medial malleolus and reaming were used in the ankle joint. The subtalar joint was not formally prepared. The TTC nail was then compressed using its internal and external compression mechanism. Finally, the calcaneal screws were inserted, first from medial to lateral using the jig. Of note, the posterior to anterior calcaneal screw was not able to be applied using the jig and was instead inserted using the perfect circle technique. Due to the supine position of the patient, the perfect circle was technically simpler to achieve by drilling from anterior to posterior. As shown in Figure 5, the midfoot osseous structures were drilled through, passing the drill bit out of the heel, then passing the screw from posterior to anterior as the drill bit was withdrawn. Final radiographs were taken (Figure 6), and the soft tissues were sutured together.

## 4. Discussion

Ankle fractures can be debilitating injuries, especially for elderly patients. Typically, these fractures are treated with open reduction and internal fixation; however, an individualized approach needs to be taken, incorporating the patient's desires, functional status, comorbidities, fracture pattern and severity, and social history. We present a case of a patient that initially refused surgery and subsequently went on to develop a severe ankle malunion secondary to noncompliance with weight-bearing precautions in her cast immobilization. Given the patient's low functional demands and social history, a shared decision was made to proceed with an operation that would allow for immediate weight-bearing that could realign her mechanical axis and lead to a pain-free limb, which ultimately can lead to an improved quality of life. Falzarano et al. compared three different treatment options (external fixation, plate and screws, or intramedullary nail) for AO 43A distal tibia fractures and evaluated baropodometric measures at 12 months. They found lateralization of the gait axis in the injured limb and increased calcaneal load [8]. If subtle changes are present in gait mechanics despite the reduction of distal tibia fractures as demonstrated by Falzarano et al., immense mechanical axis deviations such as in our patient can be expected to incur mechanical alterations along the entire limb. Georgiannos et al. performed a prospective randomized study comparing TTC nailing and traditional open reduction internal fixation (ORIF) in the management of geriatric unstable malleolar ankle fractures. Postoperative complications were four times higher in the ORIF group. The TTC nailing group had a shorter length of stay, but there was no difference in the functional outcomes of both groups, as measured by the Olerud Molander Ankle Score [14]. A recent systematic literature review by Jehan et al. in 2011 evaluated 659 TTC arthrodesis using intramedullary nails. The collective union rate was 86.7% with an average union time of 4.5 months. Complications ranged from 15% to 80% with an average of 55.7%, mostly related to stress reactions and stress fractures at the proximal nail. Reoperation rate was 22%, revision arthrodesis 3%, and amputation rate 1.5% [15].

The reduction of a chronically subluxated ankle with a syndesmotic injury can be technically challenging. The talus displaces laterally, and the distance between tibia and fibula increases, further widening the syndesmosis allowing the talus to wedge in that space [16] (Figure 2). More than one technique has been reported to help with the reduction of the talus under the tibia. In our case, we osteotomized the fibula, used thick plates and lag screws to pull the tibia to the fibula, and exaggerated a lateral start point for the bent TTC nail. Despite all these measures, the final product was still in varus. The new technique we are describing can make the reduction easier and ensure better end alignment of the ankle fusion.

In general, there are both straight and curved nails that can be used for TTC fusion. In 1962, Küntscher described a TTC fusion using an unlocked nail [17]. As the surgical procedure became accepted, companies developed nails specific for this purpose and improved nail design. In order to pass a straight nail through the calcaneus, through the talus, and into the medullary canal of the tibia, the starting point lies on the medial edge of the anterior process of the calcaneus. This is due to the ~12 degrees of physiologic hindfoot valgus with respect to the tibia. This offers less bony anchorage within the calcaneus, and thus, curved nails were designed with a built-in valgus bend to accommodate the relevant anatomy. Curved nails also permit a more lateral calcaneal entry point with decreased risk of injury to the lateral plantar nerve and vessels [10]. The valgus bend in the curved nails avoids the need for medialization of the talus and improves centralization of the nail within the medullary canal of the tibia, decreasing the risk of cortical hypertrophy, stress riser, and fracture [11–13]. A study by Marley et al. compared curved and straight designs in hindfoot nailing. They found that cortical stress reactions occur less frequently with curved nails. There were 2 cases (out of 13 cases) of stress fractures with straight nails at the level of the proximal-most tibial screw. Provided that our nail was inverted to assist with the reduction of the valgus malunion, the intended centering of the nail tip was likely altered which may lead to stress at the proximal extent of the nail. This will have to be followed over time clinically.

## 5. Conclusion

We present a case of an elderly patient with significant medical comorbidities and social issues who sustained an unstable ankle fracture that led to a severe malunion secondary with chronic lateral talus subluxation to refusal for surgery and noncompliance with weight-bearing precautions. Despite debridement of fracture callus, fibular osteotomy, and plate-assisted reduction, a displaced valgus angulation of the ankle joint remained. The nail was inserted backwards in order to use the bend in the nail as a reduction aid to finalize the reduction. We believe this technique may be of assistance to future surgeons encountering a similar situation.

## Data Availability

The supporting literature for this case report may be reviewed in the reference section.
